# A Novel Approach for Apple Freshness Prediction Based on Gas Sensor Array and Optimized Neural Network

**DOI:** 10.3390/s23146476

**Published:** 2023-07-17

**Authors:** Wei Wang, Weizhen Yang, Maozhen Li, Zipeng Zhang, Wenbin Du

**Affiliations:** 1 School of Information and Communication Engineering, North University of China, Taiyuan 030051, China; 2 Department of Electronic and Electrical Engineering, Brunel University London, Uxbridge UB8 3PH, UK

**Keywords:** gas sensor array, freshness prediction, chaotic sequence, sparrow search

## Abstract

Apple is an important cash crop in China, and the prediction of its freshness can effectively reduce its storage risk and avoid economic loss. The change in the concentration of odor information such as ethylene, carbon dioxide, and ethanol emitted during apple storage is an important feature to characterize the freshness of apples. In order to accurately predict the freshness level of apples, an electronic nose system based on a gas sensor array and wireless transmission module is designed, and a neural network prediction model using an improved Sparrow Search Algorithm (SSA) based on chaotic sequence (Tent) to optimize Back Propagation (BP) is proposed. The odor information emitted by apples is studied to complete an apple freshness prediction. Furthermore, by fitting the relationship between the prediction coefficient and the input vector, the accuracy benchmark of the prediction model is set, which further improves the prediction accuracy of apple odor information. Compared with the traditional prediction method, the system has the characteristics of simple operation, low cost, reliable results, mobile portability, and it avoids the damage to apples in the process of freshness prediction to realize non-destructive testing.

## 1. Introduction

### 1.1. Background

Apples are one of the most commonly consumed fruits by people. China’s apple production accounts for one-seventh of the world’s output, and it is an important cash crop in China. The freshness of apples is the most important indicator to evaluate the quality of apples, which directly affects the sales of apples. If the shelf life of apples can be accurately predicted, it will provide an effective guarantee for quality and output value.

Fruit and vegetable freshness prediction technology has a long history, and its freshness prediction methods [1] mainly include fuzzy sense, dielectric property, mechanical property, acoustic property, near-infrared spectroscopy, and electronic nose detection technology. Fuzzy sense mainly relies on individuals to judge the feel, smell, and experience of objects, which are highly subjective. The dielectric property is detected by using the dielectric constant of the fruit, which can be used for the detection of fruit sugar content and moisture content. Acoustic characteristics are detected using acoustic properties such as fruit reflection, scattering, transmission, and attenuation. Kinetic modeling is a technique that uses the relevant mechanical properties of fruits for testing. Near-infrared spectroscopy is the use of fruit to detect the absorption, reflection, scattering, transmission, and other characteristics of light. The above four detection methods are generally for a single detection object, which needs to be judged one by one; the detection efficiency is relatively low; and the requirements for equipment are high.

The smell of the same fruit at different growth stages is different. The odor between different varieties is also different, and the electronic nose is used to simulate the biological olfactory function to analyze and identify the odor for detection. In order to meet the requirements of rapid and non-destructive real-time monitoring of food freshness, electronic nose technology based on gas sensors has developed rapidly, and in recent years, more and more research on the freshness prediction of food has been applied by electronic nose technology.

In 2008, Antihus [2] used the PEN2 electronic nose, principal component analysis method, and LDA (Linear Discriminant Analysis) algorithm to monitor the shelf life of tomato storage, which realized the monitoring and differentiation of tomatoes with different storage times but did not realize the freshness prediction of tomatoes.

In 2013, Hui Guohua [3] proposed a storage time prediction method for Fuji apples based on electronic nose, which used the random resonance method to calculate the gas concentration data collected by electronic nose and established a prediction relationship with the storage time of apples, which detected a single apple sampling time of 3 h and a long detection time.

In 2016, Alireza Sanaeifar [4] used a low cost electronic nose to detect bananas with different shelf lives and used SVM (support vector machine) technology to predict various quality indicators of bananas, which had a good prediction effect on soluble solids and hardness but a poor prediction effect on PH and titratable acid, so that the overall freshness prediction effect of bananas did not reach the expected level.

In 2019, Wojciech Wojnowski [5] et al. proposed a prediction method for the bioamine index of refrigerated chicken based on an electronic nose. Using a modular electronic nose and a special sample chamber to analyze the volatile components of chicken and a BP neural network for data modeling, the results show that it can accurately predict the biogenic amine index of chicken. In this experiment, the biogenic amine index of chicken was predicted, but the quality of the chicken was not evaluated.

In 2020, Parthasarathy Srinivasan [6] used a self-designed electronic nose to predict the quality changes of Pacific white shrimp during storage and determined its quality by measuring PH value, determining microbial content, texture analysis, and sensory evaluation, and the identification rate of white shrimp stored at low temperatures was as high as 96.29% through the Soft-max algorithm.

In 2019, Feng Lei [7] from Jiangnan University applied electronic nose and low-field nuclear magnetic resonance technology to study the freshness of cucumbers, cherries, and tomatoes, and by monitoring their flavor characteristics and the change of moisture status, the PLS (Partial Least-Squares) algorithm model was used to predict the hardness, soluble solids, and color difference of cucumbers and tomatoes and the quality changes of cucumbers during storage. In the model, the detection cost of low-field NMR (Nuclear Magnetic Resonance) technology is high, and it is difficult to popularize and practice.

In 2020, Chen Shaoxia [8] from Nanjing Agricultural University used an electronic nose and near-infrared spectroscopy to predict the quality loss rate, VC (vitamin C) content, hardness, and other quality indicators of baby vegetables during storage. The results show that the combination of the two technologies has a good predictive effect on quality indicators. However, the combination of the two technologies increases the detection cost and makes the experimental process too complex and cumbersome. The infrared spectroscopy technology has high requirements for light source selection, which is not suitable for large-scale promotion.

In 2022, Zhang Man [9] proposed a method for predicting the freshness of cold fresh mutton based on a BP neural network using gas information and established a prediction model for the physical and chemical indicators (hardness, pH value, color, TVB-N (total volatile basic nitrogen) content) that characterize the freshness of mutton by detecting the environmental gas content. The results showed that the coefficient of prediction of physicochemical properties was above 0.9, indicating that the BP neural network has a good prediction effect. In the implementation method, the amount of data is large, and the training time is long.

In summary, through the analysis of the research status at home and abroad, the use of an electronic nose to predict the freshness of vegetables and fruits is achievable, and the detection process has the advantages of non-contact and batch detection. At present, among the relevant detection methods, there are problems such as a complex detection process, a high detection cost, an excessive amount of collected data, and a long detection time.

### 1.2. Related Work

In recent years, our research group has completed a number of studies on electronic nose freshness detection technology for apples. Guo et al. [10] built an apple freshness test platform using odor recognition technology combined with fuzzy sensory algorithms and dielectric properties to measure apple quality, and the accuracy of apple freshness determination was 93.75%. Liu et al. [11] used a self-made odor recognition system to complete a rapid evaluation of the classification of the freshness of Fuji apples. The principal component analysis algorithm was used to detect the freshness features of apples within 1 min, with an accuracy of 95.33%. Yan et al. [12] connected the smell of apples with their sweetness through the gas sensor array and realized the classification of the sweetness of apples. The CPSO-BP (BP optimization by Chaotic Particle Swarm Optimization) neural network algorithm was adopted with an accuracy of 83.33%, which was comparable to the detection accuracy of commercial near-infrared spectroscopy analyzers and realized the non-destructive testing of the sweetness of apples.

Among the existing research results [13], an odor recognition system for evaluating the freshness of Fuji apples was designed. By collecting and detecting the aroma emitted by apples, cluster analysis and a classification model are established using stable system response values. The continuous projection algorithm is used to optimize the sensor array, solve the collinearity and overlap problems, and eliminate abnormal and redundant sensors. It uses a ZigBee wireless sensor network to send data to the upward computer and uses a BP neural network algorithm optimized by the hybrid leapfrog algorithm to recognize the gas data, which improves the training speed and accuracy of the neural network. The experimental results showed that the accuracy of the method was 98.67%, and it could identify the freshness of Fuji apples quickly and comprehensively.

Apple odor information is feasible and reliable for apple quality detection, and on this basis, apple freshness prediction is further realized. Combined with the existing research results, The main work of this study is as follows: (1) This study detects the gas concentration released by apples during storage by the self-designed electronic nose system, accurately characterizes the freshness of apples by using a sensor array composed of ethylene, ethanol, oxygen, and carbon dioxide, and uses a WSN (wireless sensor network) as a means of information transmission. (2) Taking the prediction of the future freshness of apples as the starting point, the SSA optimization BP neural network added to Tent is used to further optimize the network to complete the prediction of apple odor characteristics. The Tent-SSA-BP model for apple freshness prediction is established, and finally, low cost, lossless, and efficient apple freshness prediction is realized.

## 2. Technical Principles

### 2.1. Freshness Prediction System

**Figure 1:**
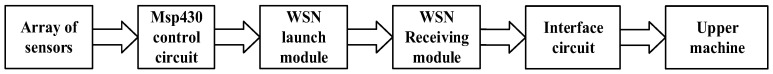
Block diagram of acquisition system.

**Figure 2:**
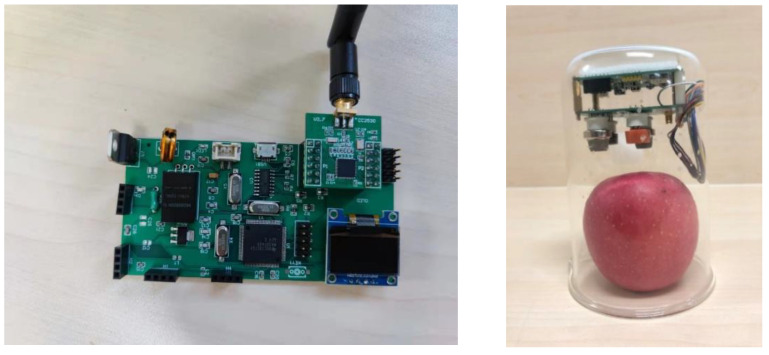
Information processing center module (**left**), Information collection (**right**).

Using a self-made dual-chip wireless acquisition and processing system, the overall function is completed by two main chips with each other, with gas concentration collection, data information processing, wireless transmission, processing result display, and the ability to work with other nodes. The block diagram of the system structure is shown in Figure 1. WSN nodes are used to realize wireless transmission during acquisition. One of the sensor arrays is connected and placed in the container where the apple is stored, and the other node is connected to the host computer to transmit data back and be processed by the host computer. Then multiple nodes can be added to form a wireless acquisition and prediction network. The actual self-made hardware is shown in Figure 2.

The sensor array is connected to the WSN transmitter module and placed in a gas environment containing the sample to be measured, which converts the gas concentration into an electrical signal packaged by the transmitter module. The receiving module is connected to the host computer, receives the data packets from the transmitting module, and uploads the data to the host computer through the serial port output for storage. The control chip of the whole acquisition system is Msp430F449, which has complete functions and can complete the preliminary data processing task. The ZigBee wireless transmission module adopts the CC2530 RF module circuit and supports the ZigBee2007Ztack protocol stack. The wireless transmission of data has the advantages of saving costs and making the system more convenient.

### 2.2. Choice of Sensor

According to the types of gases involved in the physiological action of apples after picking, the optimal sensor combination in the actual environment is selected to ensure the accuracy of gas concentration collection. According to the previous research and research results [14,15], the main response gases are selected as ethylene, ethanol, oxygen, and carbon dioxide gas sensors to form a sensor array, and the type of selection and performance indicators of the sensors are shown in Table 1. The sensor array composed of four types of sensors selected has a good response characteristic curve to the volatile gas of apples, and effectively improves the cross-sensitivity characteristics between the sensors during the detection process and the identification accuracy of apple odor in the experiment.

The advantage of using the sensor module is that after the power sensor module is turned on and the sensor module starts to work, the communication and data transmission between the main control chip can be realized through the general IO port, In terms of the collected data, the concentration value, voltage, and current value can be collected through the program according to its own needs. In addition, the use of ready-made sensor modules can also reduce the volume of the main circuit board, and you only need to reserve different voltage power supply interfaces and IO ports required for communication according to different sensor power supply needs in the design work. It also provides convenience for replacing the sensor, as the operation of the sensor ages and different environmental needs change, so it is necessary to detect and replace the sensor in time to ensure the stability and correctness of the collected data.

## 3. Algorithm Framework and Principle

The collected apple odor data is divided into a training set and a test set and imported into the optimized neural network for modeling and training, and the subsequent concentration change is predicted according to the current gas concentration of apple samples. The apple freshness is identified and classified by the predicted concentration, so as to realize the prediction of apple freshness.

### 3.1. Sparrow Search Algorithm

The Sparrow Search Algorithm (SSA) [16] is a new type of meta-heuristic algorithm. In the algorithm, individuals are divided into discoverers, watchers, and followers, with each individual corresponding to a solution. In the process of algorithmic foraging, the positions of the three are continuously updated to complete the resource acquisition. Compared with other optimization algorithms, this one is easy to implement and has relatively few control parameters and strong local search ability. In order to avoid falling into the local optimum, the tent chaotic sequence is introduced for optimization, which increases the population diversity, thereby improving the search and exploitation performance of the algorithm and increasing its stability [17].

The specific implementation steps are shown in Table 2:

### 3.2. Optimized BP Neural Network

**Figure 3:**
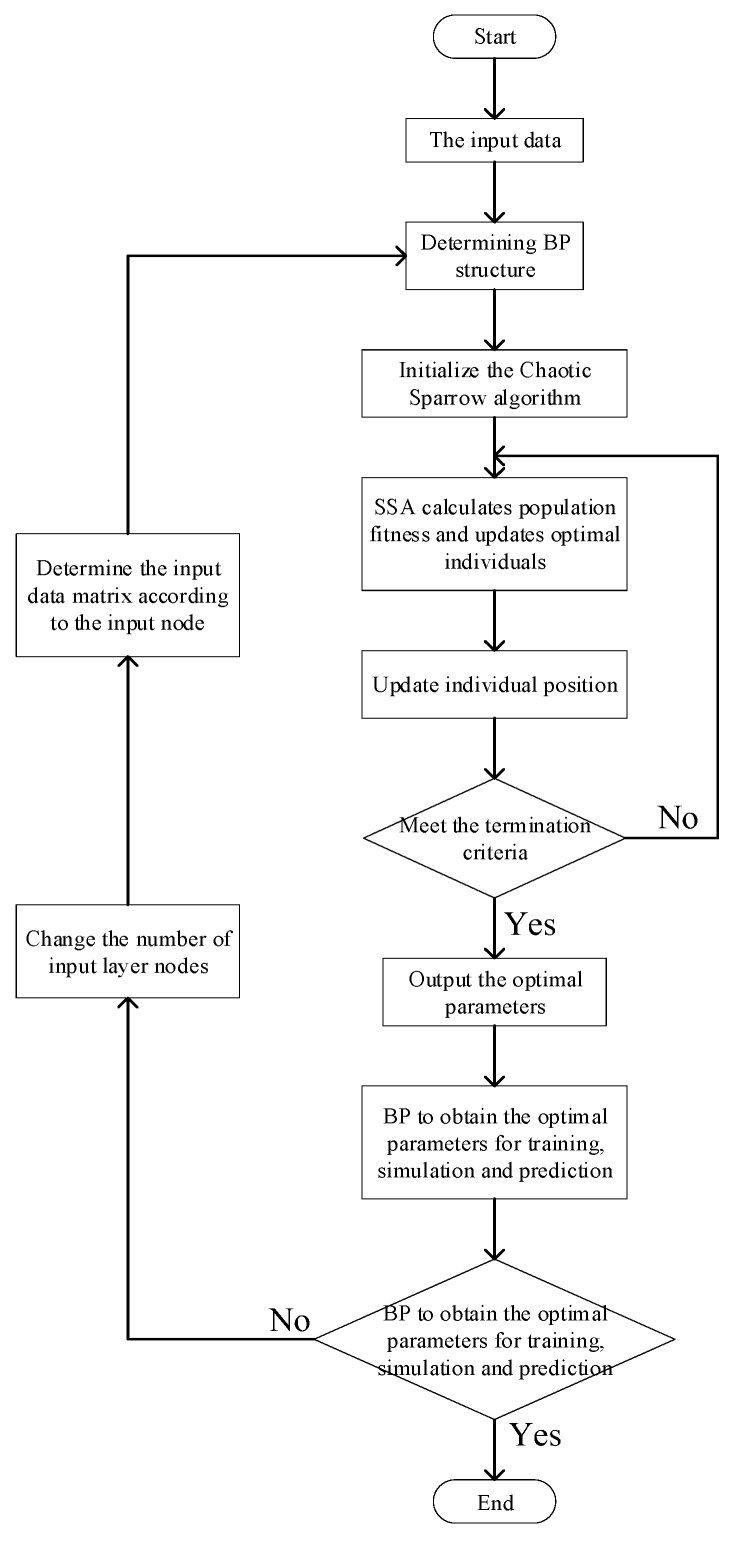
Flowchart of the algorithm.

According to the prediction of concentration change, the shelf life of apples is predicted, and the sparrow search algorithm is improved by the Tent chaotic sequence. SSA is used to optimize the BP neural network in order to complete the prediction of apple odor characteristics. Tent-SSA optimizes BP in two aspects: one is to optimize the weight threshold of BP by using the optimization function of the optimization algorithm; Second, the input layer is optimized, and the structure of the input matrix is changed by setting the expected value and the number of cycles to find the best input matrix suitable for prediction and the number of input nodes of BP, where the setting of the expected threshold is further calculated by the fitting function of the relationship between the coefficient of determination and the input vector. The optimized flowchart is shown in Figure 3.

The specific implementation steps are shown in Table 3.

**Figure 4:**
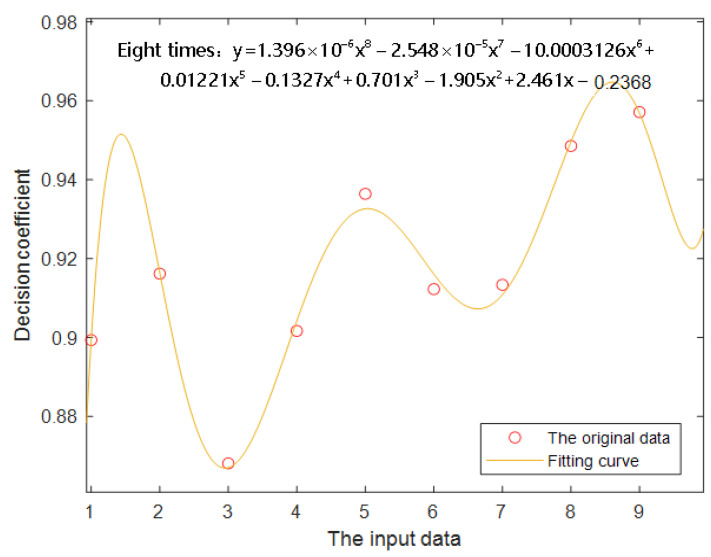
Relation diagram of fitting curve.

The pros and cons of the prediction model have a great relationship with the coefficient of determination ε_0_, and according to multiple experiments and statistical analysis of data, there is a correlation between the prediction results of the model and the arrangement of the input matrix. After a fixed number of iterations, the fitting curve between ε_0_ and the input vector obtained by multiple fitting using MATLAB is shown in Figure 4.

It can be seen from the figure that ε_0_ shows a wave upward trend with the increase in the number of input vectors. Freshness prediction is the analysis of concentration change in days, and the number of input vectors should not be too large. The relationship between derived ε_0_ and input vectors is shown in the following Equation (1), where *x* represents the number of input vectors.

(1) $$\begin{array}{ll} \varepsilon_{0}= & 1.396\times 10^{-6}x^{8}-2.548\times 10^{-5}x^{7}-0.0003126x^{6}+0.01221x^{5}\\ & -0.1327x^{4}+0.701x^{3}-1.905x^{2}+2.461x-0.2368 \end{array}$$

The goodness-of-fit degree verified by the results is above 0.96, and ε_0_ is used as the key parameter for model prediction. Through experimental testing, under indoor conditions of room temperature of 20 degrees Celsius and humidity of 50% RH, apples go from fresh to rotten in about 40 days. Each apple sample in the 800 mL container has a gas emission stability value as the characteristic value of the sample. By detecting the change in the characteristic value of apples within 40 days, a prediction model is established.

## 4. Freshness Classification Basis

In the process of storage, the physical and chemical changes of apples will lead to changes in the water content, freshness, and content and arrangement of some organic substances. The physiological tissue components inside apples can be regarded as an unconventional dielectric, and these physical and chemical changes will be further manifested in the internal biomolecules of apples in the change of charge arrangement response characteristics. Their macroscopic manifestation is the change of dielectric characteristics and its parameters, so the dielectric constant can be used to express the state of apple freshness.

Apple freshness feature detection technology based on dielectric properties has been relatively mature. In this experiment, a TH2822A handheld LCR instrument combined with a computer and a shielded box with plates was used to complete the detection of the dielectric constant of Fuji apples and classify the freshness of apples, and the relationship between the dielectric characteristics of Fuji apples and freshness is shown in the following table [10,11]. Among them, apples are divided into three categories: Fresh (apples without any wrinkles or shrinkage phenomena, not rotten); Not freshness (apples shrink after a period of time and do not decay); Decay (apples appear rotten).

## 5. Results and Analysis

### 5.1. Materials and Methods

Since the apples are picked, the cells in the fruit continue to respire, consuming oxygen while producing ethanol and carbon dioxide. In addition, ethylene is closely related to the ripeness of apples. Four gases: ethanol, ethylene, oxygen, and carbon dioxide, are selected to establish the apple freshness model. A number of Fuji apples picked from the same batch and purchased at the same time in the same market are selected and stored at 20 degrees Celsius indoors, and the volatile gas of each sample is sampled every 24 h to record the gas concentration change in the process from freshness to rot.

Based on the odor characteristics of apples, a BP neural network classification model was established. The eigenvalues of each apple sample were taken from the stable response values of the four sensors; the eigen input signal of the apple was four dimensions, and the result to be classified was three types, according to the Kolmogorov theorem combined with multiple experiments to verify that the number of optimal hidden layer nodes was 9, so the structure of the neural network was 4-9-3.

A total of 180 groups of pre-processed sample feature signals were selected, and 144 groups were randomly selected for network training. And it defined the expected output of each type of apple. For example, the expected output of a fresh apple sample is [1, 0, 0]; Not fresh is [0, 1, 0]; Decay is [0, 0, 1].

### 5.2. The Data Collection

**Figure 5:**
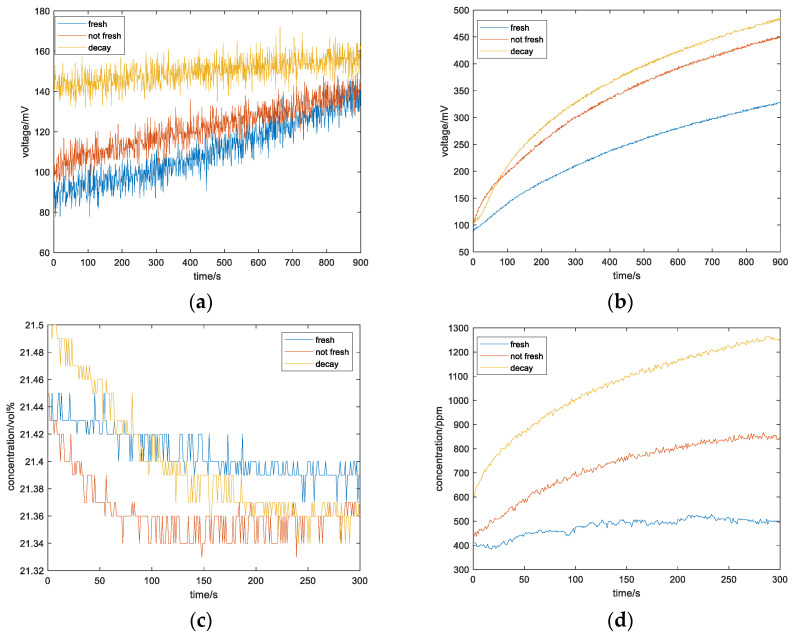
Comparison of gas concentrations of apples with different freshness: (**a**) ethylene; (**b**) ethanol; (**c**) oxygen; (**d**) carbon dioxide.

Apples of varying degrees of freshness, from fresh to rotten, are tested separately. During the detection process, apple samples and fully preheated gas sensor arrays are put into the container, one sample at a time. The odor information concentration change of each sample in the container for 15 min is collected, and the relevant gas concentration change curve detected is shown in Figure 5.

### 5.3. Data Pre-Processing

**Figure 6:**
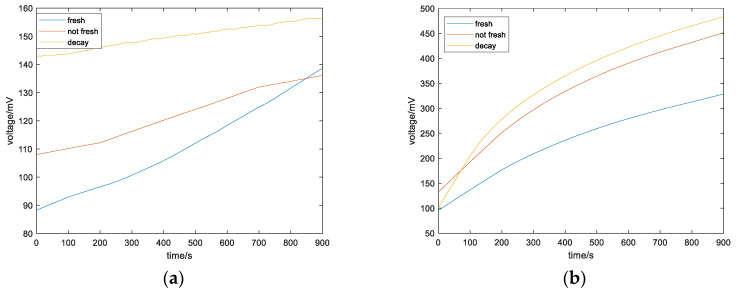

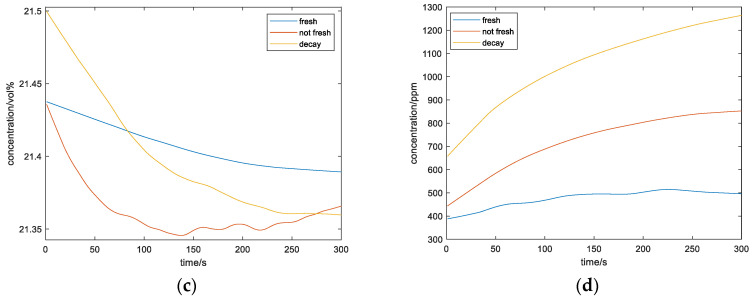
Gas concentration of apples with different freshness after filtering: (**a**) ethylene; (**b**) ethanol; (**c**) oxygen; (**d**) carbon dioxide.

As can be seen from the data in the above figure, the concentration packets of ethylene and oxygen in the collected data contain a lot of noise and need to be filtered. Linear least squares filtering is selected, and the filtering effect is shown in Figure 6.

The characteristic values of the filtered noise data are extracted, and when the gas emitted by the apple tends to be stable, the stable, average, and maximum values are taken as the characteristic signal values for statistical analysis.

### 5.4. Predicted Results

**Figure 7:**
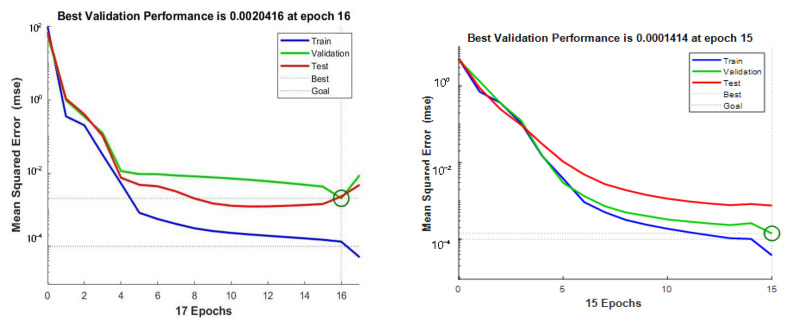
Training error before optimization (**left**), Training error after optimization (**right**).

**Figure 8:**
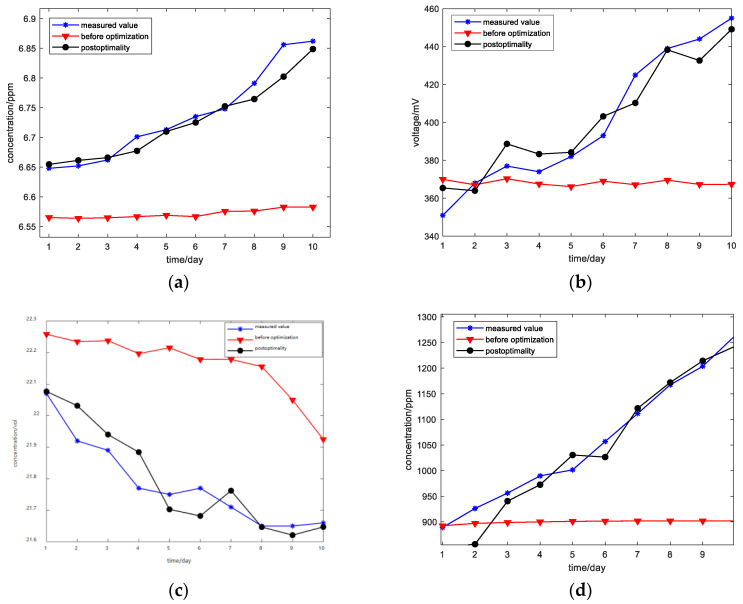
Comparison between the predicted value and the measured value of a sample before and after optimization: (**a**) ethylene; (**b**) ethanol; (**c**) oxygen; (**d**) carbon dioxide.

Figure 7 and Figure 8 show the comparison of training errors before and after BP neural network optimization.

As shown in Figure 7, the optimized neural network has a smaller prediction error. The 40-day concentration change curve of an apple sample during the detection process and the stable value concentration prediction curve of the apple sample before and after the optimization of the BP neural network are shown in Figure 8.

Based on the comparison of the above figures, it can be seen that the prediction errors of the prediction model before and after optimization are 0.002 and 0.0002, respectively, and the error after optimization is significantly reduced. The coefficient of determination is a key parameter used to reflect the reliability of model variables, and in order to evaluate the stability and reliability of the model more intuitively, the coefficient of determination is used as the evaluation index of the predictive model. Firstly, the sum of squares and total squares of the residuals are calculated according to Formulas (2) and (3), ${\mathrm{SS}}_{\mathrm{res}}$ is the sum of residual squares, $SS_{tot}$ is the total sum of squares, $y_{i}$ represents the real data, $\bar{y}$ represents the average, and $f_{i}$ is the predicted data. Then the determination coefficient is calculated according to Formula (4).

(2) $$SS_{res}=\Sigma {\left(y_{i}-f_{i}\right)}^{2}$$

(3) $$SS_{tot}=\Sigma {\left(y_{i}-\bar{y}\right)}^{2}$$

(4) $$R^{2}=1-SS_{res}/SS_{tot}$$

Finally, the coefficient of determination before optimization is 0.80057 and the coefficient of determination R^2^ after optimization is 0.95851, which shows that the prediction stability of the optimized model is better than that before optimization, the prediction error is smaller, and the performance is stable.

### 5.5. The Classification Results

**Figure 9:**
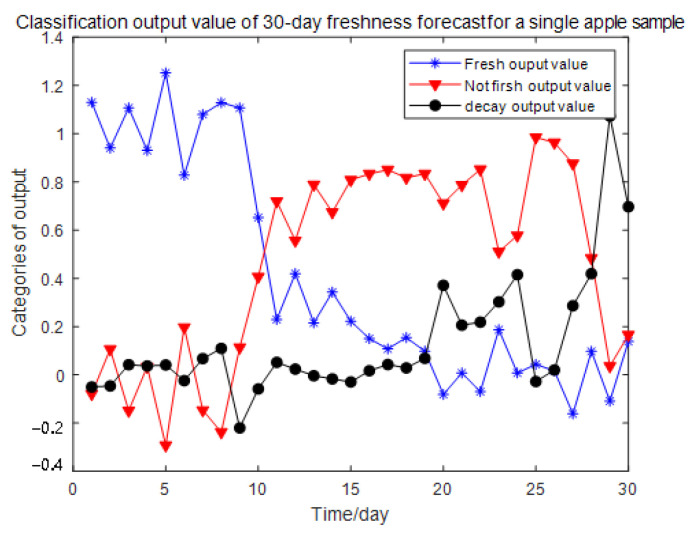
Freshness prediction output of a single apple.

Through the pre-processed 180 sets of data, the ratio of training set to test set is 8:2 to divide, of which 144 groups are used to train the classification model and the other 36 groups are shuffled and sorted for recognition verification. We obtained some specific detection results as shown in both Table 4 and Table 5, and the result change of the neural network for the freshness classification output of an apple sample within 30 days is shown in Figure 9, where the maximum value of the three types of output values on the same day is the predicted freshness result of the apple.

According to the predicted concentration, the freshness of the apples was classified and identified. By comparing the recognition results, it was found that the accuracy rate of the sixth day was the highest, and the accuracy rate was 100%. The lowest accuracy rate was 80 percent on day 30. In practical application, the prediction days can be determined according to the accuracy requirements of the freshness prediction, and the accuracy of the freshness prediction will decrease with the increase in the number of days.

According to the predicted concentration, the freshness of apples was classified and identified. Then the identification results are compared, and the accuracy rate is the highest on the sixth day, which was 100%, and the lowest on the 30th day, which was 80%. In practical applications, the number of predicted days can be determined according to the requirements for freshness prediction accuracy, and the freshness prediction accuracy will decrease as the time goes on.

## 6. Conclusions

In this paper, a gas sensor array based on a wireless transmission module is used to collect the odor information of apples with different degrees of freshness, and a system model for apple odor information prediction is established by combining deep learning algorithms and intelligent senses by using the Tent-SSA-BP neural network prediction model. Compared with existing prediction models, the experimental results show that the model has strong optimization ability, high prediction accuracy, good stability, and a coefficient of determination of more than 0.95. Combined with the apple freshness classification system based on the gas sensor array, a complete apple freshness prediction system is formed that can accurately predict the freshness of apples in the next 30 days or so with the advantages of high accuracy, low cost, a small amount of data, convenient detection, and non-destructive testing. In the next phase of research, further research will be carried out on apples under practical application conditions, such as the shelf life of apples under refrigerated conditions and the change in shelf life of apples during transportation.

## Figures and Tables

**Table 1:** Composition of sensor array.

Type of Sensor	Mainly Measured Gas	Measuring Range	Working Voltage/V
MQ3	ethanol	(25~500) × 10^−6^ ppm	5.0
MG811	Carbon dioxide	(0~10,000) × 10^−6^ ppm	6.0
ME2-O2	oxygen	0~25% Vol	3.3
ME3-C2H4	ethylene	(0~100) × 10^−6^ ppm	5.0

**Table 2:** Tent-SSA implementation steps.

Step Number	Step Content
1	Set the population size N, the number of discoverers $p_{num}$, the number of reconnaissance warnings $s_{num}$, the target function dimension D, the initial values of the upper and lower bounds ub and lb, respectively, the maximum number of iterations T, and the solution accuracy ε.
2	Tent is applied to generate N D-dimensional vectors *Z_i_*, and within the range of values of the variable by the carrier $X_{new}^{d}=d_{\min}+(d_{\max}-d_{\min})Z_{i}$, *d*_min_ and *d*_max_ are, respectively, the minimum and maximum values of the d-dimension vector $X_{new}^{d}$.
3	Calculate the fitness $f_{i}$ and select the optimal fitness $f_{g}$, the worst fitness $f_{w}$, and the corresponding positions xb and xw, respectively.
4	Select the first $p_{num}$ with good fitness to be the discoverer, and the rest as joiners; update the locations of the discoverer and joiner.
5	Randomly select $s_{num}$ as the vigilant and update their position.
6	One iteration was performed to calculate the fitness $f_{i}$ and average fitness $f_{avg}$ for each animal. Perform an iteration to calculate the fitness and average fitness of each animal
7	If $f_{i}\geq f_{avg}$, the individual is chaotically disturbed, if the individual’s performance is better after disturbance, it will replace the previous individual; otherwise, it will remain unchanged.
8	If $f_{i}<f_{avg}$, mutation(x)=x(1+N(0,1)) was used to conduct Gaussian mutations on individuals. *x* represents the original parameter value, *N*(0,1) represents the normally distributed random number, and mutation(*x*) is the value after Gaussian mutation.
9	Update the optimal position xb and its fitness and the worst position xw and its fitness in the entire population.
10	Determine whether the maximum number of iterations or solution accuracy is reached, and if so, the loop ends and outputs the optimal parameter result.
11	If not, go back to Step 6 and iterate again.

**Table 3:** Optimized BP implementation prediction steps.

Step Number	Step Content
1	Initialize. According to the input matrix, determine the BP topology, initialize the maximum number of iterations T0, and determine the coefficient of determination ε.
2	Use an optimized sparrow search algorithm for iterative optimization through Tent.
3	The SSA algorithm is completed, the optimal parameters are output, and the values are assigned to the BP network for prediction and calculate ε.
4	According to $\begin{array}{ll} \varepsilon_{0}= & 1.396*10^{-6}x^{8}-2.548*10^{-5}x^{7}-0.0003126x^{6}+0.01221x^{5}\\ & -0.1327x^{4}+0.701x^{3}-1.905x^{2}+2.461x-0.2368 \end{array}$, calculate the quality threshold value $\varepsilon_{0}$ in this prediction result.
5	Make a judgement. If $\varepsilon <\varepsilon_{0}$, adjust the input matrix and return to Step 1 until the number of iterations T0 is reached or Step 6 is satisfied.
6	Make a judgement. If $\varepsilon >\varepsilon_{0}$, the loop ends, and the predicted result is output.

**Table 4:** The relationship between the dielectric properties and the freshness of Fuji apples [10,11].

Improvement of Characteristics	$\mathrm{The}\;\mathrm{Equivalent}\;\mathrm{Capacitance}\;C_{S}/e^{-10}F$	$\mathrm{Loss}\;\mathrm{Factor}\;D/e^{-2}$	$\mathrm{Relative}\;\mathrm{Dielectric}\;\mathrm{Constant}\;\varepsilon /e^{-1}$
fresh	2.0–2.5	6.1–6.8	5.0–5.5
not fresh	1.2–2.0	4.5–5.8	3.5–5.0
decay	>0.8	>2.8	>2.8

**Table 5:** Partial prediction results of an apple.

Time/d	The Actual Freshness	The Classification Results of Neural Network Output			Predicted Results
		Fresh	Not Fresh	Decay	
6	Fresh	0.8272	0.1970	−0.0242	Fresh
10	Fresh	0.6521	0.4064	−0.0585	Fresh
15	Not fresh	0.2219	0.8077	−0.0297	Not fresh
20	Not fresh	−0.0814	0.7105	0.3708	Not fresh
30	Decay	0.1381	0.1651	0.6967	Decay

## Data Availability

Publicly available datasets were analyzed in this study. This data can be found here: s2005131@st.nuc.edu.cn (W.Y.).
